# Two-year efficacy after first transscleral controlled cyclophotocoagulation in patients with and without pseudoexfoliation

**DOI:** 10.1007/s00417-021-05157-5

**Published:** 2021-04-02

**Authors:** Markus Lenzhofer, Melchior Hohensinn, Wolfgang Hitzl, Veit Steiner, Armin Motaabbed, Karolina Motloch, Hans Peter Colvin, Herbert A. Reitsamer, Sarah Moussa

**Affiliations:** 1 Department of Ophthalmology and Optometry, Paracelsus Medical University, Salzburger Landeskliniken, Muellner Hauptstrasse 48, 5020 Salzburg, Austria; 2 Research Program Experimental Ophthalmology and Glaucoma Research, Paracelsus Medical University, Muellner Hauptstrasse 48, 5020 Salzburg, Austria

**Keywords:** COCO, Controlled transscleral cyclophotocoagulation, CPC, PEX, Efficacy

## Abstract

**Purpose:**

Transscleral controlled cyclophotocoagulation (COCO) is a transscleral 810-nm diode laser cyclophotocoagulation that automatically adjusts the applied laser energy utilizing an optical feedback loop. The present study investigates the influence of pseudoexfoliation (PEX) on the efficacy of COCO in a Caucasian study population.

**Methods:**

Retrospective data from 130 consecutive eyes were analyzed during a 2-year follow-up. Baseline characteristics, intraocular pressure (IOP), number of IOP-lowering medications, visual field, best-corrected visual acuity (BCVA), and secondary surgical interventions (SSI) were analyzed. The primary endpoint was IOP reduction at M24 compared to baseline, and the secondary endpoints were IOP course, reduction of IOP-lowering medications, surgical success, and IOP-lowering SSIs stratified by PEX and baseline IOP.

**Results:**

IOP reductions of −35, −39, −25, −25, −23, −34, and −36% could be achieved from baseline to D1, W1, M1, M3, M6, M12, and M24 (all *p* < 0.001), respectively, while there was a significant overall reduction over time (*p* < 0.001) in the number of topical IOP-lowering medications postoperatively. The proportion of eyes requiring additional systemic IOP-lowering medication reduced from 31 to 0% at M24 (*p* = 0.025). Eyes without PEX and IOP < 30 mmHg at baseline had the lowest risk for IOP-lowering SSIs (*p* < 0.03). BCVA dropped at M12 (0.25 [95% CI: 0.12–0.38]), and the drop persisted during the following 12 months.

**Conclusion:**

The present study demonstrates a midterm IOP-lowering effect after COCO while reducing the burden for topical and systemic IOP-lowering medications. Patients without PEX and IOP < 30 mmHg have a lower risk of SSI. The procedure per se cannot be excluded as causative for the decreased postoperative BCVA. Further prospective investigations are suggested.

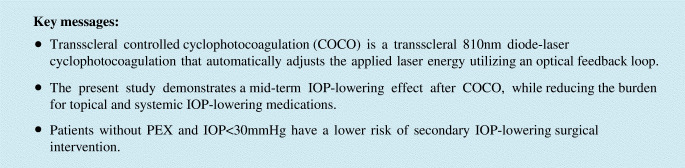



## Introduction

Glaucoma is one of the leading causes of irreversible blindness in the world. Much effort has been made to develop surgical techniques for lowering intraocular pressure (IOP) to treat glaucoma [1–5]. Aqueous outflow obstruction is the main cause of IOP elevation, which can be mitigated either by increasing outflow or reducing aqueous humor production [6]. Although newly introduced minimally invasive glaucoma surgery and traditional filtering glaucoma surgery (both aim to increase aqueous outflow) are performed in rising numbers [7–9], cyclodestructive procedures have their significance in the daily clinical routine and are applied frequently. Cyclodestructive procedures target and destroy the ciliary body epithelium to lower intraocular pressure by reducing aqueous humor inflow into the eye [6]. A second mechanism for postoperative IOP reduction is the enhancement of uveoscleral outflow by increasing scleral permeability [10]. The most commonly used approach is transscleral laser cyclophotocoagulation (tCPC) [6], as first described by Beckman in 1972 [11, 12]. It can be performed using neodymium:yttrium-aluminum-garnet (Nd:YAG) or a diode laser [13, 14]. Currently, the evidence is still inconclusive as to which type of cyclodestructive procedure is superior [10, 15].

Although tCPC is described as a safe procedure in numerous case series, the following serious postoperative complications have been reported in the literature: pain, transient conjunctival burn, severe iritis, prolonged inflammation, hyphema, hypotony, phthisis bulbi, atonic pupil, cataract progression, loss of vision, and sympathetic ophthalmia [6, 11, 12, 15].

Traditionally, tCPC is indicated in patients with refractory glaucoma, after failed filtering glaucoma surgery, in patients with limited best-corrected visual acuity (BCVA), in patients with no visual potential in the need of pain relief, and in patients with complicated glaucoma or scarring from previous surgeries [6, 10, 15]. More recently, the indication for tCPC has been extended to patients with non-refractory glaucoma and good vision [12].

Efficacy has been shown [6, 13, 15], but it remains unclear if tCPC results in better outcomes than other glaucoma treatments (e.g., Ahmed valve) [15]. Furthermore, the risk factors for surgical failure are not yet clear. Due to the potential side effects and reduced individual predictability of success, tCPC procedures are usually considered as second-line therapy [6, 15].

**Fig. 1:**
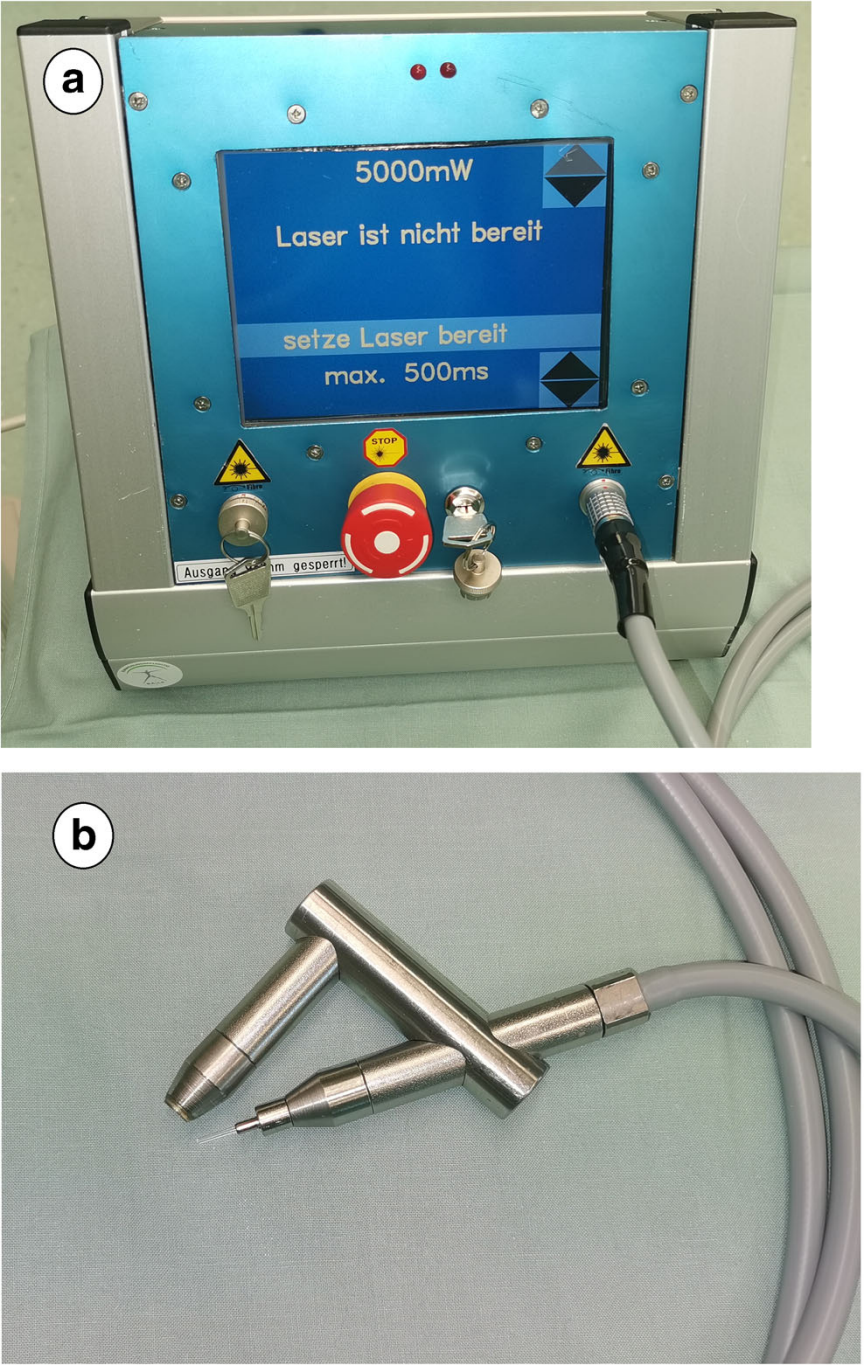
Transscleral controlled cyclophotocoagulation (COCO). Controlled cyclophotocoagulation is a newer transscleral 810-nm diode laser cyclophotocoagulation method to lower intraocular pressure in glaucoma treatment. The machine consists of a stationary box (**a**) with integrated laser, computer and touch displays, and a solid handpiece (**b**). The laser energy is delivered through a glass fiber probe (small extension) while touching the conjunctiva and measured with the inbuilt sensor (big extension) anterior the cornea. It automatically adjusts the applied laser energy by an optical feedback loop

Controlled cyclophotocoagulation (COCO, Preussner, Mainz, Germany; Fig. 1), introduced in 1996, is a newer transscleral 810-nm diode laser tCPC method that automatically adjusts the applied laser energy by an optical feedback loop [16, 17]. A small fraction of the impinged laser radiation passes the ciliary body after multiple scattering. Subsequently, it is reflected from the fundus, exiting the eye through the optical pathway, and is recorded by a photodetector touching the cornea [16]. The detected signal changes during progressive coagulation of the ciliary body tissue and thereby triggers a shutoff of the laser, preventing overspilling energy delivery at the point of adequate ciliary body coagulation [18]. This reduces the postoperative inflammation and occurrence of pops (i.e., intraocular uveal microexplosions) [16, 18].

There is evidence that optic nerve damage is more pronounced in pseudoexfoliation (PEX) eyes compared to primary open-angle glaucoma at the time of diagnosis. The response to medical therapy is poorer [19]. The responses to argon laser therapy and filtering surgery seem to be roughly the same in these two types of glaucoma [19]. The prevalence of PEX is reported to be between 0.2 and 30% depending on the study population [20, 21]. Efficacy data on COCO and the effects of PEX on tCPC, in general, are rare. Therefore, the present study reports the efficacy and influence of PEX on the efficacy of COCO treatment in a Caucasian study population with non-refractory and refractory glaucoma.

## Methods

The study and data accumulation were carried out with the approval of the local ethics committee (Ethikkommission Land Salzburg). The study was in adherence to the tenets of the Declaration of Helsinki and conforms to the ethical standards of the local ethics committee. The study was conducted in a university health care setting in a catchment area, where another public facility to get glaucoma surgeries is more than 60 km off-site. In this retrospective, single-center, clinical cohort study, consecutive eyes with non-refractory and refractory glaucoma treated with COCO, a CE-marked prototype, which was developed by Preussner (Mainz, Germany), were enrolled. A chart review collected data of the first two postoperative years: the baseline demographic data included the type of glaucoma and PEX status, postoperative IOP assessed by single Goldmann Applanation tonometry measurements in all patients, number of topical and systemic IOP-lowering medications, mean deviation of the visual field (MDVF, SITA standard 30-2, HFA II, Humphrey Instruments, USA), BCVA, and secondary surgical interventions (SSI, if applicable) were recorded. SSIs included consecutive IOP-lowering procedures (IOP–SSI) after the initial COCO (e.g., second COCO, other tCPC, filtering glaucoma surgery, and selective laser trabeculectomy [SLT]) and cataract surgery. Exclusion criteria were a lack of follow-up and baseline BCVA less than counting fingers. To assess BCVA, decimal BCVA in charts was converted to LogMAR, as previously described by Holladay [22].

Refractory glaucoma was defined as uncontrolled intraocular pressure with evidence of optic nerve and/or visual field deterioration despite maximally tolerated topical and/or systemic antiglaucoma medications, failed IOP-lowering surgical treatment, or combined surgery and medicines. Non-refractory glaucoma was defined as uncontrolled intraocular pressure with evidence of optic nerve and/or visual field deterioration despite maximally tolerated topical and/or systemic antiglaucoma medications without a history of IOP-lowering surgical treatment (excluding small laser treatments, e.g., selective laser trabeculoplasty [SLT], argon laser trabeculoplasty [ALT], and argon laser peripheral iridotomy [ALPI]). In a few cases, uncontrolled IOP included normotensive patients whose glaucoma was progressing rapidly and whose IOP could not be lowered further by other means.

Two-hundred twenty-eight consecutive eyes of 202 patients received the first COCO between 2010 and 2013 according to the Mainz Protocol described by Preussner [16, 18].

To summarize, 16 effects were applied under analgosedation with standard settings (5 W and maximum exposure time 0.5 s; Fig. 1) after mydriasis and marking the ciliary body with diaphanoscopy [18].

The postoperative treatment regimen included the initial continuation of topical IOP-lowering medication, discontinuation of systemic IOP-lowering medication, and a low dose of topical corticosteroids for a few days (Dexamethasone TID for 5–7 days). Topical steroids and IOP-lowering medications were adapted and tapered off rapidly depending on the grade of postoperative inflammation and IOP. The indication and time of SSI were a clinical decision based on the discretion of the surgeon (e.g., did not reach target pressure and progression).

Visits were categorized at baseline (BL), day 1 (D1), week 1 (W1), and at months 1 (M1), 3 (M3), 6 (M6), 12 (M12), and 24 (M24) postoperatively. Data was therefore pooled into bins. For the analysis of SSIs, the exact time to SSI was used for analysis in Kaplan–Meier and Cox proportional hazard models.

The primary endpoint was IOP reduction at M24 compared to BL. Secondary endpoints were IOP reduction and change in the number of IOP-lowering medications at the remaining postoperative visits, BCVA, and MDVF change at M12 and M24, and the time to IOP-SSI stratified by PEX and IOP.

According to the definition by the World Glaucoma Association [23], all eyes were classified in the following two groups based on their last available postoperative visit: surgical failure and surgical success. In the case of an increase in the number of medications compared with BL, relative IOP reduction < 20% compared to baseline, the presence of an IOP-SSI (except for standard cataract surgery), loss of visual acuity to light perception acuity or worse, or a postoperative IOP of <6 mmHg at the last visit, the patient was classified in the surgical failure group. Surgical success was defined as a lack of the above criteria plus relative IOP reduction ≥ 20% compared to BL. Surgical success was further characterized according to whether it was achieved without (complete success) or with and without IOP-lowering medications (qualified success).

For reporting purposes, significant ocular hypotony was defined as IOP < 6 mmHg, present at two consecutive visits postoperatively >30 days apart.

### Statistical methods

The endpoints were set before analysis began and were not altered. Data were checked for consistency and normality. Generalized estimation equation (GEE) models were used to analyze continuous data over time, and Holmberg–Bonferroni adjusted *p* values were computed for pairwise comparisons. Independent and dependent tests were used to analyze data. Kaplan–Meier and Cox proportional hazard models were used to test PEX, refractory glaucoma, age, and IOP at baseline for proportions of reoperation. Proportionality assumption was tested using the Cox model, and McNemar’s test for dependent proportions was utilized to compare the number of IOP-lowering medications over time. Ninety-five percent confidence intervals for means were computed and illustrated using Whisker plots. All reported tests were two-sided, and *p* values < 0.05 were considered statistically significant. All statistical analyses in this report were performed by the use of STATISTICA 13 (StatSoft, Tulsa, OK) and PASW 24 (IBM SPSS Statistics for Windows, Version 21.0., Armonk, NY).

## Results

One hundred seven right eyes (47%) and 121 left eyes (53%) of 103 female (46%) and 125 male (54%) patients were legible. In total, 228 consecutive eyes from 202 patients underwent surgery. Thirty-nine (17%) and 59 (26%) were excluded from the analysis due to lack of follow-up or baseline BCVA less than counting fingers, respectively. After applying the inclusion and exclusion criteria, 130 eyes were enrolled.

Baseline demographic data can be seen in Table 1. Forty eyes (30%) were classified as refractory glaucoma, while 90 eyes (70%) were treated for non-refractory glaucoma. In 43 eyes (33%), PEX was present. There was no significant difference in IOP or number of IOP-lowering medications with or without PEX in baseline data (*p* = 0.86 and 0.44).

**Table 1:** Baseline demographic data, IOP course, and course of IOP-lowering medications

Mean ± SD, *n* = 130		
Age	67.7 ± 18.6	
Best corrected visual acuity (LogMAR)	0.44 ± 0.32	
Mean deviation in the visual field (dB)	−13.3 ± 4.33 (range −16.4 to −10.3)	
	**IOP raw values**	**IOP reduction**
all *p* < 0.001^*^		
IOP baseline (mmHg)	24.0 ± 7.9	compared to baseline
IOP 1 day (mmHg)	15.5 ± 5.3	−35%*
IOP 1 week (mmHg)	14.6 ± 5.0	−39%*
IOP 1 month (mmHg)	18.1 ± 8.7	−25%*
IOP 3 months (mmHg)	18.0 ± 6.9	−25%*
IOP 6 months (mmHg)	18.5 ± 7.6	−23%*
IOP 12 months (mmHg)	15.9 ± 6.9	−34%*
IOP 24 months (mmHg)	15.4 ± 4.3	−36%*
	**Medications raw values**	**Medications reduction**
Medications: *p* value (time) < 0.001**		
Medications baseline	3.59 ± 1.16	compared to baseline
Medications 1 day	3.08 ± 1.06	−14%**
Medications 1 week	3.31 ± 1.04	−8%**
Medications 1 month	3.37 ± 1.2	−6%**
Medications 3 months	3.07 ± 1.16	−14%**
Medications 6 months	3.11 ± 1.32	−14%**
Medications 12 months	3.06 ± 1.18	−15%**
Medications 24 months	3.13 ± 0.96	−13%**

*/**indicates a statistical significance *p* < 0.05 generated by GEE models and based on Holmberg–Bonferroni adjustments of *p* values for multiple comparisons, standard deviation (SD), and medications: IOP-lowering medications: *p* value (time) < 0.001**

Forty-three eyes (33%) had PEX glaucoma, 41 eyes (32%) had primary open-angle glaucoma, 11 eyes (8%) had normal-tension glaucoma, 8 eyes (6%) had neovascular glaucoma, 5 eyes (4%) had chronic angle-closure glaucoma, 4 eyes (3%) had pigment dispersion glaucoma, 3 eyes (2%) had congenital glaucoma, 1 eye (1%) had aphakic glaucoma, and 14 eyes (11%) had secondary glaucoma due to other reasons (e.g., silicone oil-induced glaucoma, post-traumatic glaucoma, and post-keratoplasty glaucoma; Table 2).

**Table 2:** Surgical history of eyes receiving transscleral controlled cyclophotocoagulation

Procedure*	Percentage (counts)
No surgical IOP-lowering pretreatment	55% (71)
SLT	19% (25)
ALT	2% (2)
ALPI	1% (1)
Trabeculectomy	22% (29)
tCPC	9% (9)
Ahmed glaucoma valve	3% (4)
Bearveldt glaucoma shunt	1% (1)
Cypass	2% (3)
XEN	2% (2)

This table shows the intraocular pressure-lowering procedures performed before the first transscleral controlled cyclophotocoagulation in 130 eyes. *One eye may have had multiple procedures. Non-refractory glaucoma (*n* = 91, 70%) was defined as eyes, which have received no intraocular pressure-lowering procedures excluding small laser treatments (e.g., selective laser trabeculoplasty [SLT], argon laser trabeculoplasty [ALT], and argon laser peripheral iridotomy [ALPI]). Eyes, which have received trabeculectomy, transscleral cyclophotocoagulation (tCPC), tube shunts, or minimally invasive glaucoma surgery (e.g., Cypass or XEN gel stent implantation) were classified as refractory glaucoma (*n* = 39, 30%).

**Fig. 2:**
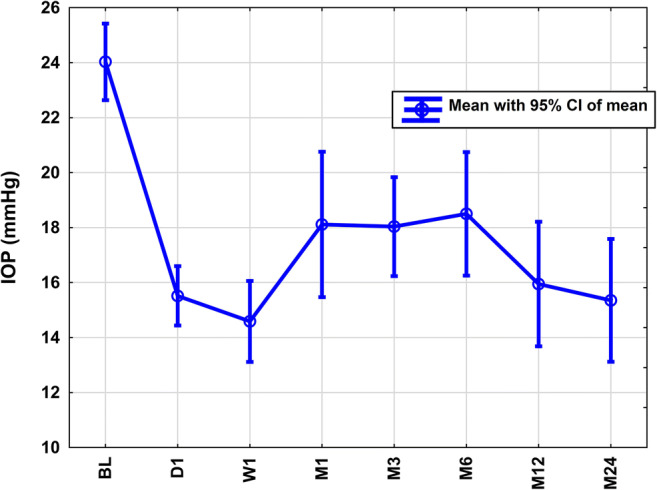
Intraocular pressure (IOP) course in the first 24 months after transscleral controlled cyclophotocoagulation. Each postoperative visit showed a statistically significant reduction of intraocular pressure of −35, −39, −25, −25, −23, −34, and −36% (all *p* < 0.001) compared to baseline (BL)

At each postoperative visit, a significant reduction (all *p* < 0.001) of mean IOP was achieved after COCO (Fig. 2, Table 1). There was no significant difference in the postoperative IOP courses when stratified by PEX (*p* = 0.24).

**Fig. 3:**
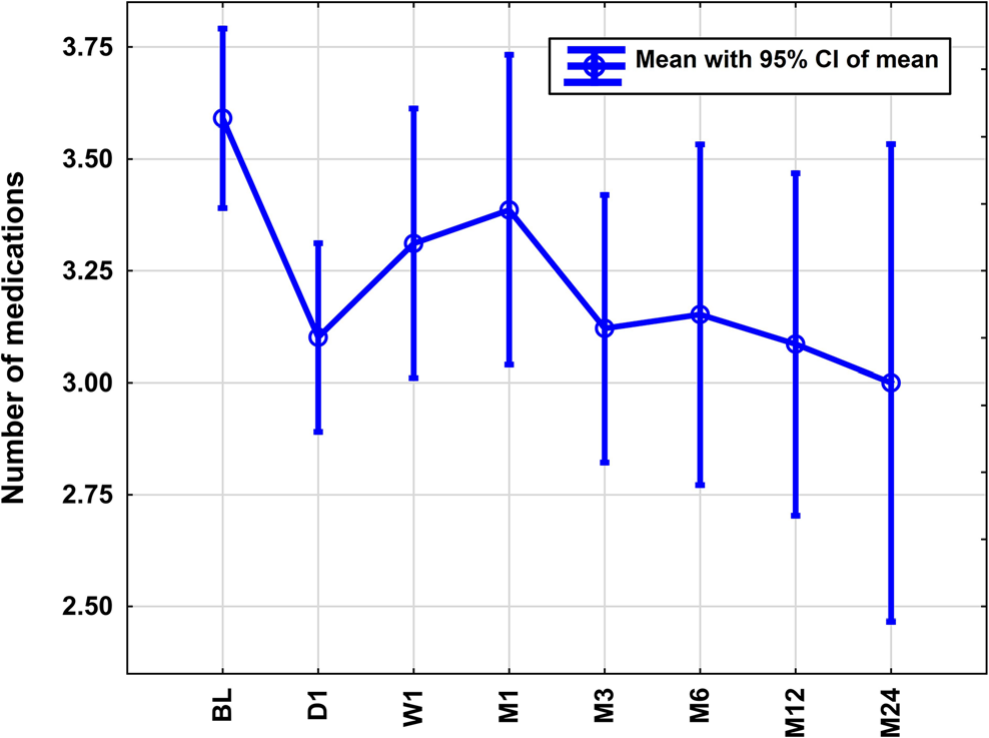
Course of a number of topical intraocular pressure (IOP)-lowering medications in the first 24 months after transscleral controlled cyclophotocoagulation. There was an overall reduction of topical IOP-lowering medications after transscleral controlled cyclophotocoagulation (*p* < 0.001). Further, the proportion of patients requiring additional systemic IOP-lowering medications reduced from 31 to 0% at 24 months (*p* = 0.025)

We found a statistically significant overall reduction (*p*[time] < 0.001; Fig. 3) in the number of topical IOP-lowering medications from baseline to D1 (*p* = 0.001), M3 (*p* = 0.006), M12 (*p* = 0.019), and M24 (*p* = 0.004). The proportion of eyes requiring additional systemic IOP-lowering medication dropped form 31 at BL to 0% at M24 (*p* = 0.025). There was no difference in the postoperative courses of topical IOP-lowering medications stratified by PEX (*p* < 0.09).

**Fig. 4:**
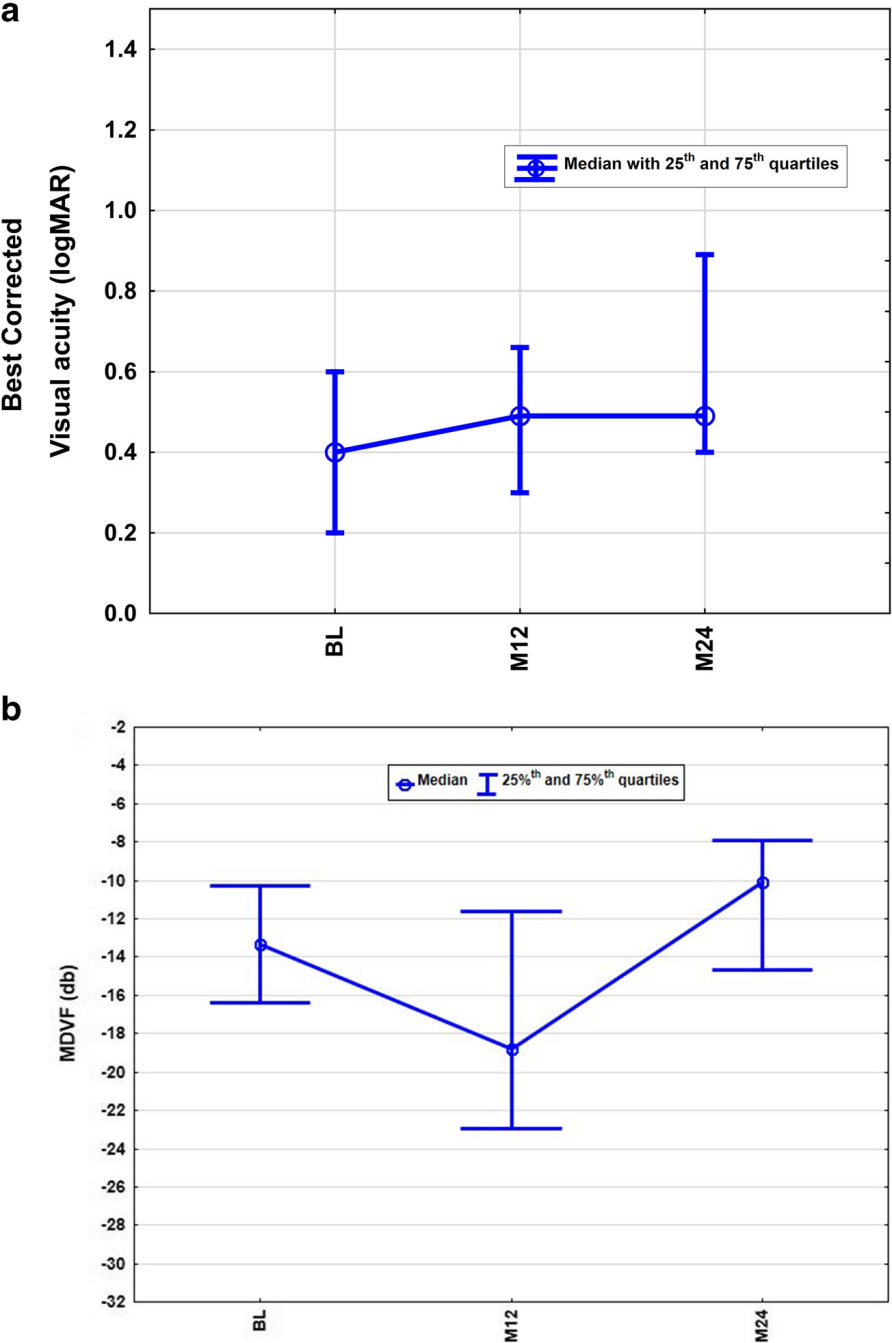
Course of best-corrected visual acuity (BCVA, **a**) and median deviation of visual field examination (MDVF, **b**) of patients receiving transscleral controlled cyclophotocoagulation. Significant changes of median BCVA (**a**; baseline 0.40 [25th quartile: 0.20, 75th quartile 0.60], M12 0.49 [25th quartile: 0.30, 75th quartile 0.66; *p* < 0.001], and BCVA drop persisted at M24 0.49 [25th quartile: 0.40, 75th quartile 0.89; *p* = 0.76]). MDVF (**b**) did not change significantly over time (*p* = 0.17)

No case of phthisis bulbi or hypotony was recorded (0%). Median BCVA dropped at M12 (baseline 0.40 [25th quartile: 0.20, 75th quartile 0.60] and M12 0.49 [25th quartile: 0.30, 75th quartile 0.66]; *p* < 0.001), and the drop persisted during the following 12 months (M24 0.49 [25th quartile: 0.40, 75th quartile 0.89]; *p* = 0.76, Fig. 4a). There was no change of MDVF over 2 years (Fig. 4b; *p* = 0.17).

### Secondary surgical intervention (SSI)

**Fig. 5:**
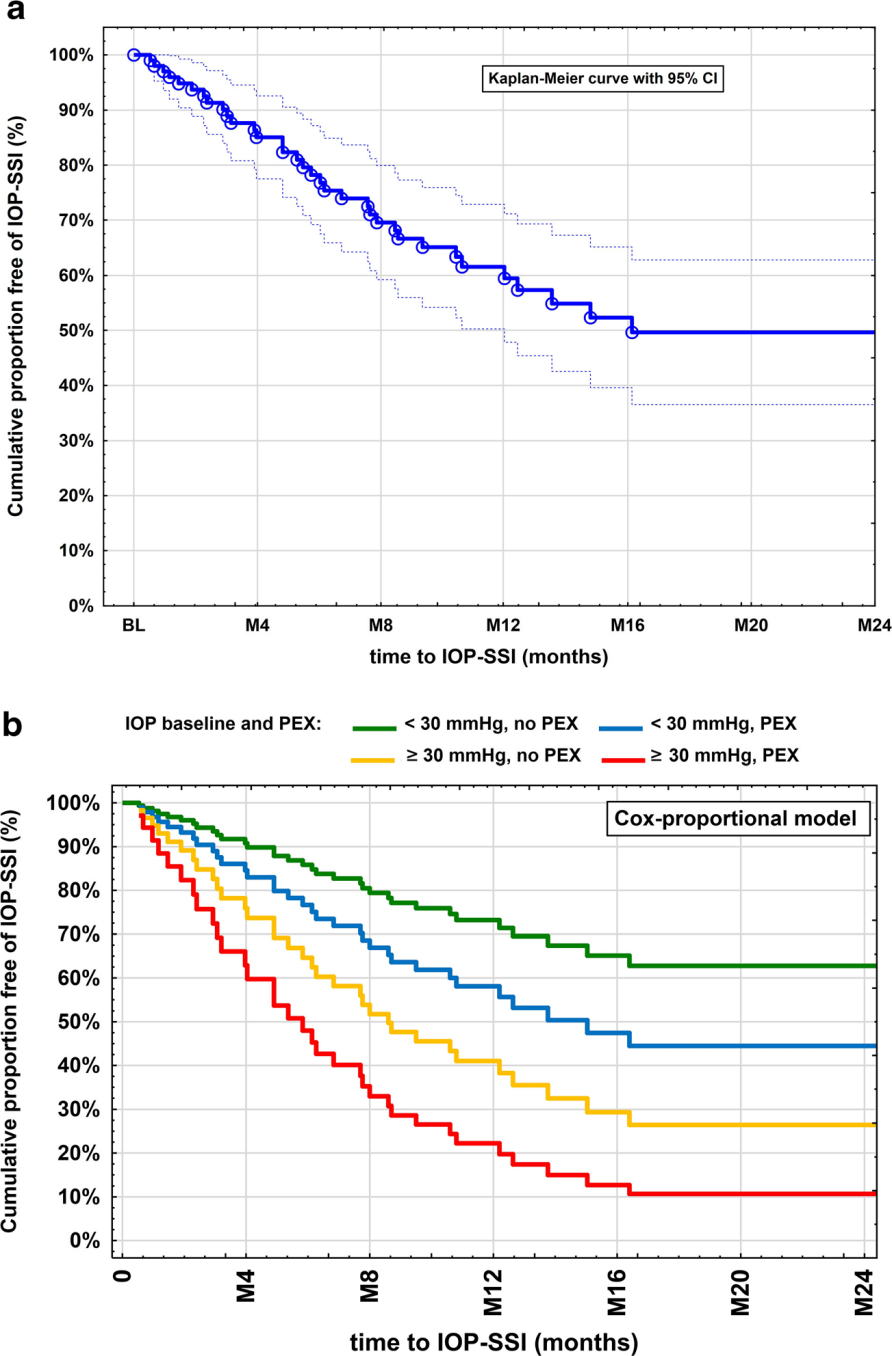
Kaplan–Meier curves for IOP-lowering secondary surgical intervention (IOP–SSI) after transscleral controlled cyclophotocoagulation (COCO) in a Caucasian study population (**a**) and stratified by intraocular baseline pressure and PEX (**b**). **a** Cumulative proportion of eyes without IOP–SSI after COCO. **b** Results with taking pseudoexfoliation (PEX) and intraocular pressure (IOP) into account (Cox proportional hazard model)

The median time to IOP–SSI was 492 days (95% CI: 221–762 days; Fig. 5a). A stratification by baseline IOP (< versus ≥30 mmHg; IOP ranged from 11–52 mmHg; 26/130 [20%] had an IOP ≥ 30 mmHg) combined with PEX (presence versus absence) revealed that eyes with an IOP ≥ 30 mmHg with PEX (HR 4.81 [1.06–21.8]; *p* = 0.03) and without PEX (HR 2.85 [95% CI: 1.13–7.22]; *p* = 0.005) and PEX eyes with an IOP < 30 mmHg (HR 1.74 [95% CI: 0.8–3.77]; *p* = 0.026) had a significantly higher risk for IOP–SSIs compared to non-PEX eyes with IOP < 30 mmHg (Fig. 5b). Age was not found to significantly influence time to IOP–SSI (*p* = 0.38). Table 3 gives an overview of all IOP–SSIs. Seven eyes (5%) had cataract surgery after COCO and before any potential IOP–SSI. Refractory glaucoma showed no difference in SSIs compared to non-refractory glaucoma (*p* = 0.20).

**Table 3:** Secondary surgical intervention (SSI) after transscleral controlled cyclophotocoagulation within 24 months

	*N*	%
No SSI	71	52
Re-COCO*	49	36
Phacoemulsification and IOL implantation	7	5
tCPC*	3	2
SLT*	3	2
Trabeculectomy*	2	1
Cypass*	1	1
XEN*	1	1
Ahmed*	0	0
ALT*	0	0
ALPI*	0	0
Baerveldt*	0	0

This table shows all SSIs performed after the first transscleral controlled cyclophotocoagulation (COCO) in 130 eyes. *Intraocular pressure-lowering lowering SSI. Selective laser trabeculoplasty (SLT*), classic transscleral cyclophotocoagulation (tCPC*), XEN gel stent implantation (XEN*), argon laser trabeculoplasty (ALT*), and argon laser peripheral iridotomy (ALPI*).

### Surgical success and failure

Fifty-six (43%; 95% CI: 34.4–52.0%) eyes were classified as a qualified success, while just two (2%; 95% CI: 0.2–5.4%) eyes were classified as complete surgical success at the last available postoperative visit. Seventy-four (57.0%, 95% CI: 48.0–65.6%) eyes were classified as surgical failure, while of these, forty-nine (66%) were classified as a failure due to a secondary COCO. A surgical failure occurred in 23/43 (53.5%) in patients with PEX and 51/87 (58.6%) without PEX. No significant difference was found (*p* = 0.71). A surgical failure occurred in 21/40 (52.5%) in patients with refractory glaucoma and 53/90 (58.9%) without refractory glaucoma. No significant difference was found (*p* = 0.57).

## Discussion

TCPC is currently considered the gold standard of cyclodestructive procedures in glaucoma treatment and is commonly applied in eyes with severe glaucoma and limited visual prognosis that are unresponsive to other interventions [24]. In recent years, newer methods like endoscopic cyclophotocoagulation, ultrasound cyclodestruction, and controlled tCPC (COCO) have been adopted in clinical routine [25]. During the COCO procedure, an optical feedback mechanism steered by a computer decreases the risk of excessive or insufficient energy delivery to the ciliary body. Indications are increasingly no longer limited to refractory glaucoma in clinical care. Efficacy data is rare for the newer method COCO. In our study, we found an overall substantial and midterm IOP-lowering effect following COCO treatment. There was also a significant reduction of both topical (−13% at M24) and oral glaucoma medications (31→0%) during follow-up. The absence of PEX and lower baseline IOP had a lower risk of IOP–SSIs.

Our study is unique for several reasons: Firstly, it evaluates the largest population on the efficacy of COCO (except Preussner’s data [16–18], who invented the procedure). Secondly, it is the first study to assess the influence of PEX on efficacy after COCO.

### Efficacy

Our midterm COCO results are in line with the literature reporting efficacy of classic tCPC [6, 10, 15, 24–29]. In a retrospective analysis investigating tCPC, Kosoko et al. [30] reported a 44% reduction of IOP in mean after 19 months. Their postoperative IOP was 20.3 ± 8.7 mmHg at the last available visit. In another large retrospective study allowing multiple tCPC treatments, a reduction of IOP from 34.1 ± 10.6 to 20.1±9.3 mmHg was reported [26], while the mean number of IOP-lowering medications dropped from 2.3 to 1.7 in this study [26].

In literature, Micropulse CPC showed a reduction of IOP between 17.2 and 21.6% sustained for up to 6 months [31, 32]. Whether COCO is superior effective compared to Micropulse CPC cannot be concluded with our data and is suggested to be investigated in a randomized prospective trial.

Our results confirm Preussner’s (developer of COCO) statements [16–18]. We were able to show a −36% IOP (to 15.4 ± 4.3 mmHg) reduction, while the number of topical IOP-lowering medications was reduced by −13% (to 3.13 ± 0.96) at 24 months. Here, we wish to point out that these results were accompanied by discontinuation of systemic IOP-lowering medications (e.g., acetazolamide) in all patients on systemic IOP-lowering medications (31→0%). This is of great importance considering that the possibility of removing, e.g., acetazolamide, sometimes poses a treatment goal in itself due to the poor long-term tolerability of the drug.

The definitions of success and SSIs are highly variable among different studies, limiting the comparability of results [24, 33–36]. In the study of Kosoko et al., 62% of the patients had an IOP reduction of >20%, while 52% met these criteria and an absolute IOP lower than 22 mmHg [30]. In contrast to our study, most other studies allowed for multiple tCPCs to achieve surgical success [33–35, 37]. Due to the study design, we excluded further data when an IOP–SSI (including second COCO) was recorded. This was done for the reason that cyclodestructive procedures ideally should reach their treatment goal within the first procedure, and we aimed to study the effect of the first COCO. Furthermore, as in filtering glaucoma surgeries, re-performing the same operation (e.g., in another quadrant) is usually considered an SSI.

Although the association of re-treatment and energy delivery in classic tCPC remains unclear, Ishida et al. reported some evidence of higher re-treatment rates in modest energy application compared to lower re-treatment rates in higher energy application during tCPC procedures. Although COCO is a tCPC method, where less energy is applied compared to classic tCPC [16, 18], this study shows clinically relevant, prompt, and long-lasting IOP reduction and reduction of medications after COCO. A lower energy application may reduce the complication rates (e.g., phthisis), whereas it may raise the likelihood of repeating the procedure to reach the treatment goal in classic tCPC [24]. Both the phthisis and ocular hypotony rates were very low (both 0%). This would support our hypothesis that the inbuilt feedback mechanism of COCO helps reduce side effects by reducing energy delivery while retaining efficacy compared to classic tCPC [15, 24–29]. Since COCO controls the delivered energy by a feedback loop and, therefore, the applied energy is very low, this might elevate the risk to re-treat. Re-treatment rates (38%) and IOP–SSI after COCO were comparable to other retrospective studies after classic tCPC [30, 38]. However, multiple treatments were necessary to reach higher success rates in some studies [26, 27]. After tCPC, particularly in younger patients, IOP–SSI rate was higher [39]; this was not the case for COCO in the present study.

The range of indications for cyclodestructive procedures is recently expanding in literature so that these treatment options are becoming available to patients with less severe disease and better overall prognosis [25]. We would like to point out that it remains unclear whether classic filtering procedures or COCO are more effective as a primary treatment option. Both refractory and non-refractory glaucoma were investigated in this study. We found no influence on efficacy parameters, especially IOP–SSIs, between these two groups.

The present study delineates the influence of PEX and baseline IOP on the rates of SSIs. Patients with the absence of PEX had a lower risk for reoperation. This should be taken into consideration when indicating COCO in PEX patients.

### Pseudoexfoliation (PEX)

The high number of patients with PEX treated with COCO was to be expected since the PEX syndrome is very common in our catchment area, as it is also common in the Scandinavian countries [40]. Furthermore, this rate conforms to the rate of PEX patients receiving filtering glaucoma surgeries in our study center (data not shown). In the present study, the absence of PEX was shown independently to lower the risk for IOP–SSIs after COCO, although no effect of PEX on the IOP course or course of a number of IOP-lowering medications could be detected. This may be explained by the retrospective study design. PEX glaucoma is usually associated with very high IOPs. We confirm that IOP at baseline is an independent risk factor (*p* = 0.024); however, please note that PEX was also found as an independent risk factor (*p* = 0.017). So Fig. 5 not only provides information of patients <30/≥30 mmHg but also differentiates between patients with or without PEX and illustrates the corresponding effects. We here have to point out that the absence of PEX means a heterogeneity of glaucoma types (everything except PEX glaucoma).

Our IOP–SSI data is very robust since we are the only hospital in our catchment area offering surgical intervention for treating glaucoma. During the years of the recruitment period, COCO procedures accounted for approximately 5% of all glaucoma surgeries in our study center. Overall—in contrast to classic tCPC literature [24, 41]—PEX seems to negatively influence efficacy in terms of IOP–SSI after COCO in the same way as PEX may hinder laser uptake in endoscopic CPC [42]. The finding that PEX is a risk factor for more IOP-SSIs can be explained by the fact that PEX causes chronic progressive congestion of the outflow pathways of the eye by protein material. When the IOP starts to rise in PEX patients, it is due to the increased outflow resistance. According to the Goldmann equation, aqueous inflow and aqueous outflow are the most important factors influencing IOP. Cyclodestructive procedures reduce aqueous inflow. In eyes where the outflow resistance cannot be decreased by bypass or other concepts, or conversely in eyes where the outflow resistance is increased drastically and progressively due to PEX, multiple cyclodestructive procedures must be applied with caution since the equilibrium of aqueous inflow and outflow can easily be destabilized. This may increase the risk for postoperative hypotony or phthisis after multiple cyclodestructive procedures. Further prospective investigations are suggested.

### Complications

The primary outcome measures in this study were midterm efficacy parameters. A detailed safety analysis was not attempted due to the retrospective study design. However, no (0%) case of ocular hypotony was reported during the study period. Furthermore, no case of phthisis bulbi was reported. Given the large number of eyes in this study, at least some cases of phthisis or ocular hypotony might have been expected. The surprising lack of these adverse outcomes might be explained by less energy being applied during the procedure by way of the feedback mechanism, previously described, that is specific to COCO [16, 17].

As published in several studies, after tCPC [12, 24, 37, 38, 43], we also found a significant decrease of BCVA after COCO at the 1-year follow-up. Literature about BCVA course after tCPC is not consistent since other studies did not find any change of BCVA after tCPC [44, 45]. As in the study of Rasmuson et al. [24], the decrease of BCVA after COCO occurred within the first postoperative year. We affirm the findings of Rasmuson et al. that the relatively low proportion of patients with primary open-angle glaucoma compared to other studies might explain BCVA decrease [44, 45]. We would also like to mention that a decrease in BCVA might occur after filtering glaucoma surgeries [46]. Besides disease severity of the investigated patient population and comorbidities (e.g., in neovascular glaucoma), the procedure per se cannot be excluded as causative for the decreased postoperative BCVA.

MDVF, on the other hand, was not found to significantly change during the follow-up after COCO. A comparison of our MDVF data against tCPC literature is not possible due to the lack of substantial data on visual field courses after classic tCPC.

Large, well-designed randomized controlled trials are therefore needed.

### Limitations

This study is limited by its retrospective design. IOP, medications, BCVA, and MDVF courses were assessed retrospectively. Therefore, GEE models were used to compute results. In general, GEE models perform well when few observations of each subject are recorded and can also further model non-normal responses. A further limitation of the study is that the included study population was heterogeneous (non-refractory and refractory glaucoma and different types of glaucoma). This has been addressed by careful consideration of this fact during data analysis. Thirdly, we compared refractory and non-refractory glaucoma but did not include filtering glaucoma surgeries as a control group. Fourthly, IOP values are based on one measurement. This poses a risk of regression to the mean which represents a possible bias to better results [24]. On the other hand, suboptimally adjusted IOPs in the disease course might lead to more frequent IOP measurements and therefore confound results with regard to negative findings. Therefore, further prospective randomized investigations on efficacy and safety after COCO are suggested.

In conclusion, this is the first study to describe the influence of PEX on efficacy outcomes after COCO. The present study clearly demonstrates the midterm efficacy of COCO and shows a lower rate of reoperations when PEX is absent in lower baseline IOPs within a Caucasian patient population. Hypotony rates are very low after COCO. The procedure can be a suitable interventional treatment option for many patients with glaucoma, although a potential postoperative decrease of BCVA has to be considered for this procedure.
